# Integrated care: a new model for dental education

**DOI:** 10.1038/s41415-021-3318-z

**Published:** 2021-08-13

**Authors:** Asha Thomson, Andrew J. Dickenson, Maria Ross-Russell

**Affiliations:** 1 Senior Leadership Fellow, NHSE East of England, UK; 2 Regional Postgraduate Dental Dean, Health Education England, UK; 3 Deputy Postgraduate Dental Dean, Health Education England, UK

## Abstract

The COVID-19 pandemic has had a devastating health, economic and social impact on the UK health services. The learning from redeployment demonstrated that dental professionals can be rapidly integrated into the wider healthcare system, but the challenge is how this can be sustained in the future. This is an opportunity for dental training to be incorporated into a more integrated model of care, and this article outlines a collaboration between NHS England and NHS Improvement, Health Education England and Local Dental Networks to establish a novel training opportunity. This Assistant Dentist Integrated Care Pilot Programme has provided retention of dentists within areas of unmet need and introduced innovative opportunities for dental workforce transformation.

## Key points

- Local workforce opportunities may improve recruitment and retention within the dental profession.
- Innovative 'outside the box' thinking is crucial to develop healthcare, including dentistry.
- Collaboration is key, not just between stakeholders but also between dentistry and wider health and social care.

## Background

The global repercussion of the COVID-19 pandemic has had a devastating health, economic and social impact, which continues to disrupt the sustainable delivery of health services. During the first wave of the pandemic, dental professionals were redeployed into frontline positions to provide clinical care normally identified as outside their usual scope of practice. The well-documented integration of dental personnel into the wider health system was a remarkable achievement and, consequently, practitioners acquired new clinical, leadership and management skills. The learning from the pandemic has demonstrated that dental personnel can be integrated into the wider system, rather than being on the periphery of mainstream healthcare, but the challenge is how this can be maintained in the 'new normal'. This should be viewed as a positive opportunity for dental training to be incorporated into a more integrated model of care, and this paper outlines a pilot collaboration between NHS England and NHS Improvement (NHSE/I), Health Education England and Local Dental Networks, which has established a novel educational programme that crosses primary and secondary care boundaries. Rather than focusing on a resumption of previous service models, this crisis affords an opportunity to rethink the future of dentistry and address identified system-level failures, which is the basis for emerging dental transformation strategies.^1^

During the first wave of the COVID-19 pandemic there was significant engagement from the dental profession in response to regional and national redeployment requests. Nationally, over 2,000 dental team members applied for redeployment, 900 from within the East of England. This was in line with the Office of the Chief Dental Officer guidance: 'Redeploying the clinical dental workforce to support the NHS clinical delivery plan for COVID-19'.^2^ Through the successful redeployment of foundation dentists during the initial surge of activity, the concern of lack of understanding of the skills held by the dental profession in relation to the wider health and social care system was highlighted. The importance of integrating oral health on a global scale within healthcare systems has become an increasingly prominent topic of discussion within dentistry, although the remaining challenge is how this can be expanded through promotion of closer interprofessional working across the health and social care systems.

The understanding of global oral health and the utilisation of the transferable skills the dental profession holds is an important aspect of delivering an integrated healthcare system to focus on the inclusion rather than the exclusion of oral health.^3^ To develop a collaborative workforce that can integrate into the wider healthcare system and encourage patient-centred healthcare models, an innovative Assistant Dentist Integrated Care Pilot Programme was conceived based on published learning from the pandemic. The authors understand this is the first initiative of its kind, linking primary and secondary care services, education and training into a single funded programme.

The pilot was established across the East Anglia region through collaboration between NHSE/I, the East Anglia's Local Dental Network and Health Education England. The concept was initiated to support the recruitment and retention of general dental practitioners (GDPs) into areas of unmet need, to serve the local population with regards to both oral and general health, while ensuring interprofessional working and collaboration remained a sustainable strategic workforce priority. In line with the NHSE/I Dental Transformation Strategy (2020-2022), the requirement to move towards a flexible model of care delivery, addressing prevention and reduction in health inequalities, and supported by a wider dental skills mix is described and evidenced in the pilot outcomes.

This article highlights the importance of integrated healthcare, interprofessional working, and the need to provide innovative opportunities to the future dental workforce in order to incorporate dentistry within the wider health and social care system to create a sustainable and effective role as a dentist. It is also proposed that to encourage staff retention within areas of unmet need requires innovative training opportunities, funding and collaborative workforce transformation planning.

## Collaboration and change

Prior to the COVID-19 pandemic, the Local Dental Network, supported by NHSE/I and Health Education England, had collaborated on various initiatives to retain and recruit workforce within areas of unmet need. The social media age of 'Instagram dentistry' was becoming increasingly evident as a prominent influence for the early-career dentists, their career motivations and their geographical preferences when deciding on areas to live and work.^4^

**Fig. 1:**
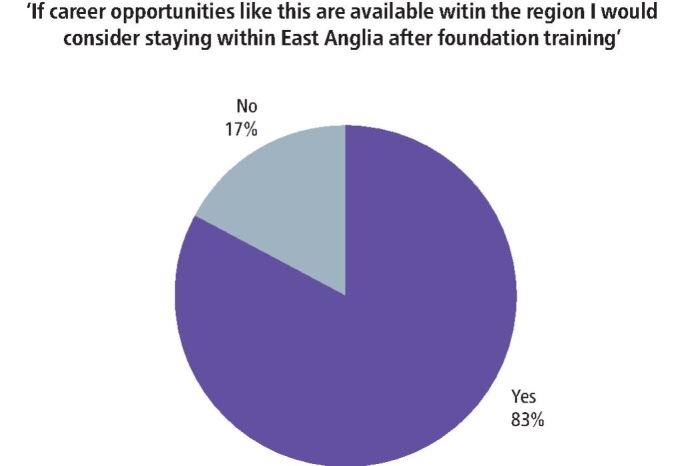
Data from survey

Change and collaboration was already underway before the COVID-19 disruption and an upskilling programme was in development. This programme would allow for a dentist to work in primary care dentistry as an associate, with an additional opportunity to be part of an upskilling programme. The proposed pilot was originally linked to oral surgery services due to the increased availability of upskilling opportunities across primary and secondary care. Data were collected to support and provide evidence for a case of need for upskilling programmes, based on workforce questionnaire surveys. Stakeholder engagement events promoted the scheme, targeted at foundation dentists, which identified their specific learning needs (summarised in Figure 1).

Figure 1 highlights that 83% of respondents answered 'yes' when asked if they would consider staying within East Anglia if career opportunities such as the proposed pilot oral surgery upskilling programme were available within the region. Although the programme will now be altered with regards to the placement component, interest in innovative opportunities is clear. From the 17% (6) of respondents who answered 'no', the reasons given were:

- Four respondents advised that due to family and marital commitments, they would be unable to stay within East Anglia
- One respondent advised 'staying within East Anglia does not interest me'
- One respondent advised 'this programme sounds great but I would prefer to be closer to London'.

The trainees were also asked the question: 'What interests you about the proposed programme?' The most common response was 'the mix of hospital and practice'. This feedback identified a need for change, challenging the opportunities and pathways currently available to dentists at the early stages of their career.

Following multiple engagement events and securing interest for the programme from both potential host practices and dentists, COVID-19 struck and the cessation of routine dental treatment halted primary care dentistry. Regional leadership was at the forefront of the dental profession to ensure that access to urgent dental services was available for patients in need.

To adopt a truly transformational approach and implement change, a collaborative working style has been identified to ensure that our aspirational changes can be put forward, with the patients' needs and best interests at the heart of any decisions made. In order to align with the NHS Long Term Plan, workforce transformation must acknowledge the requirement for having sufficient workforce with the correct skills, capable of delivering care to patients now and in the future.

This change has been embedded within the NHS strategy and has enabled change, with the opportunity to integrate dentistry further within the healthcare system across the East of England.

## Opportunities and optimism

Providing opportunities and creating optimism within the dental workforce can provide a two-way benefit to embed the dental workforce deeper within health and social care. As the COVID-19 pandemic affected the capacity of the original upskilling programme due to constraints on existing dental services and oral surgery services in both primary and secondary care, an opportunistic approach was taken to reinvent the programme in correlation with the COVID-19 pandemic.

**Fig. 2:**
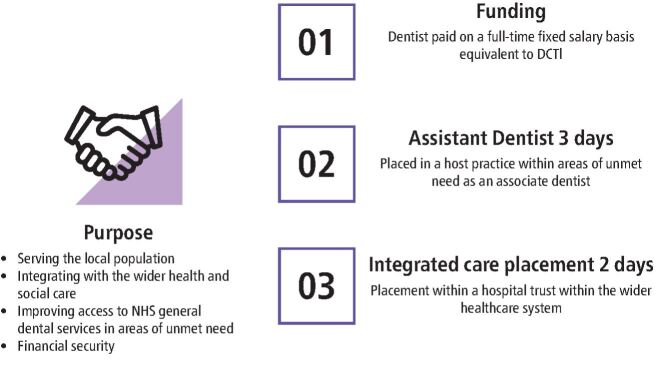
Assistant Dentist Integrated Care Pilot Programme model Composite © lushik/DigitalVision Vectors/Getty Images Plus

The upskilling programme was converted to the fully funded Assistant Dentist Integrated Care Pilot Programme through a collaborative approach between Health Education England, NHSE/I and the Local Dental Network, with the implementation of the pilot outlined in Figure 2:

- Raise awareness among primary care dental practitioners of the key healthcare strategic drivers, including the NHS Long Term Plan
- Improve access to NHS dental services within areas of high need
- Decrease strain on existing services
- Provide beneficial assistance within hospital trusts during the COVID-19 pandemic
- Support dentists in the early years of their career post-foundation dental training
- Enhance interprofessional and collaborative working
- Aim towards utilising the transferable skills of dentists within the wider health and social care sector in areas of need within East Anglia
- Act as a pilot programme within East Anglia's areas of need to utilise the skillset of GDPs not only within primary dental care but also across the wider health and social care system.

## Versatility and vision

The transferable skillsets which the dental profession possess are a unique aspect that can be utilised to enhance the healthcare system. Dental care professionals work within a variety of settings including primary care services, community services and secondary care services, and they may also hold non-clinical and educational roles.

Interprofessional working is a continuation from interprofessional education, and with the transferable skillset of a dentist, the need for future emphasis to be placed on utilising the versatility of the dental workforce and integration has been highlighted during the COVID-19 pandemic. As September approached and the foundation dentists who had an unpredicted beginning to their career aimed to secure job roles, many dentists had anxiety about job prospects and some practices felt under pressure to recruit an associate dentist due to the financial strains from the pandemic.

Utilising the medical knowledge of the dental profession with the enhanced technical skills and abilities highlights the versatility of the skillset held by the dental profession and enhances the vision of a collaborative workforce within healthcare. Instead of the phrase 'healthcare and dentistry' being used, healthcare should include dentistry as highlighted by the World Health Organisation: 'Oral health is a key indicator of overall health, wellbeing and quality of life'.^5^

The proposal of the pilot programme would allow dentists to be employed on a fully funded basis with an equivalent salary to those who are within dental core training 1 posts. After foundation training, dentists may opt to continue within formal training programmes; however, the reasons for those that do not follow this route are unknown. Often, many trainees are unable to travel or change their geographical locations for training posts and this may highlight why some dentists may voluntarily select not to follow the training pathway; however, why should these dentists lack opportunities within the GDP pathways? This question was raised and so the pilot programme was formed on the model below.

## Ideas and innovation

Innovation within healthcare systems is crucial to initiate change. With the COVID-19 pandemic affecting the original programme due to the capacity of upskilling placements, ideas were developed to create this innovation of an integrated care pilot which the authors believe is the first of its kind.

Through collaboration with the hospital trusts, the potential rotations during the secondary care placement include but are not limited to:

- Occupational health (OH) - to work with the team on immunisation and vaccinations, fully supported and trained by OH-trained nurses
- Flu programme - to work with the flu team to vaccinate staff and patients, fully supported and trained by an OH flu nurse
- Emergency department - to work with the emergency department team to undertake observations, assessments and general care. The dentists were under the supervision of a senior nurse in the emergency department - this also allows the dentist to contribute to oral health problems with which patients may present
- Acute medical unit - observing and assessment of patients
- Integration within medical ward rounds to gain a deeper insight into multidisciplinary team working and hospital care for patients
- Project development to link secondary and primary care and/or incorporate oral health initiatives
- Experience with oral and maxillofacial surgery/orthodontic units within the trusts
- Incorporation of dental upskilling within the programme with relation to periodontology, endodontics and oral surgery.

## Direction and discovery

The pilot is a unique collaboration across the healthcare system, combining clinical experience with blended education and training. The premise was directed by the COVID-19 pandemic, which identified the benefits of adaptive leadership and innovation. This pilot supports the multiple work streams as part of NHSE/I East of England's Dental Transformation Strategy 2020-2022. This is an initial step to foster links between dentistry, primary care networks and integrated care systems through a shared learning objective.

**Fig. 3:**
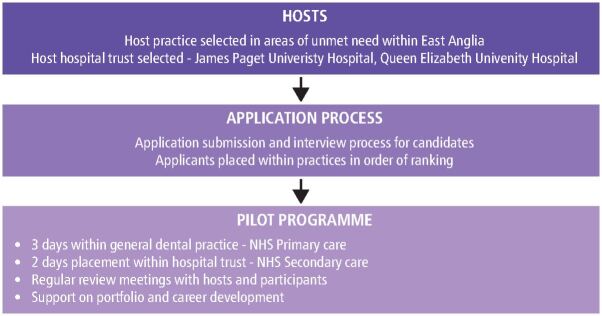
Pilot process

The implementation process of the pilot programme is highlighted in Figure 3.

On completion of the recruitment process, geographical location was identified as the most significant barrier. After a competitive interview process, five dentists were appointed based on their identified learning needs and enthusiasm to work differently.

## Discussion and conclusion

This novel programme has been delivered using a combination of clinical and academic work-based placement, similar in structure to existing dental training models. It offers a unique opportunity for GDPs wishing to enhance their clinical skills within primary care while developing additional transferable skills through interprofessional working within secondary care. This model differs from dental core training, which offers specific skill acquisition within a single setting and is clearly defined within an agreed curriculum. The integrated care model allows dentists to gain a range of skills aligned to their personal needs, identified through a robust personal development plan, with the added benefit of flexibility and self-directed learning (Table 1).

**Table 1:** Intended outcomes

The overall intended outcomes of the programme	
Improvement in dental access within areas of unmet need	Increase retention and/or recruitment of dentists in areas of unmet need
Improvement in interprofessional working	Establishment of access to dental services in areas with poor access
Integration of dentistry within the wider health and social care system	Utilisation and development of the transferrable skillset within dentistry
Enhancement and upskilling of the dental workforce to meet the needs of the local population	To provide development opportunities to GDPs within primary care

A full evaluation will be commissioned when the pilot is completed, to evaluate its long-term benefits and evidence for future funding bids. Additional outcome measures would include qualitative and quantitative data collected at the end of the pilot, including surveys and interviews from candidates, multi-source feedback from both the host and practice, patient feedback questionnaires for candidates, data collected with regards to the experience of hosting a dentist and the retention of dentists within the region.

On the predicted success of the pilot, there would be the opportunity to potentially branch out the programme further to additional aspects of health and social care, with enhanced opportunities to incentivise recruitment and retention of the dental workforce. This could result in the improvement of dental access in areas of unmet need, as well as offering a suite of additional training openings aligned to portfolio careers but without the requirement of a formalised training programme.

This pilot programme has demonstrated that interprofessional collaborative working is achievable. It is inexpensive, provides additional clinical access in areas of high demand and enhances the clinical skills of GDPs. In addition, it has allowed dentists to develop knowledge, skills and understanding of the healthcare system, which is not covered within the undergraduate or foundation curriculum. It is proposed that integrating dentistry into a wider healthcare system supports workforce retention in areas of poor dental access, transforms access to clinical care and promotes career development opportunities.

COVID-19 has emphasised both the importance of oral health and the unique challenges faced across dentistry. To sustainably embed dentistry's valuable contribution across the health and social care system, innovative thinking should be supported to ensure that the profession continues to be integrated and positively evolve.
